# Clinical, Pathological and Virological Outcomes of Tissue-Homogenate-Derived and Cell-Adapted Strains of Porcine Epidemic Diarrhea Virus (PEDV) in a Neonatal Pig Model

**DOI:** 10.3390/v16010044

**Published:** 2023-12-27

**Authors:** Carlos López-Figueroa, Esmeralda Cano, Núria Navarro, Mónica Pérez-Maíllo, Joan Pujols, José I. Núñez, Júlia Vergara-Alert, Joaquim Segalés

**Affiliations:** 1 Unitat Mixta d’Investigació IRTA-UAB en Sanitat Animal, Centre de Recerca en Sanitat Animal (CReSA), Campus de la Universitat Autònoma de Barcelona (UAB), 08193 Bellaterra, Barcelona, Spain; carlos.lopez@irta.cat (C.L.-F.); esmeralda.cano@irta.cat (E.C.); nuria.navarro@irta.cat (N.N.); monica.perez@irta.cat (M.P.-M.); jpujols1816@gmail.com (J.P.); joseignacio.nunez@irta.cat (J.I.N.); 2 WOAH Collaborating Centre for the Research and Control of Emerging and Re-Emerging Swine Diseases in Europe (IRTA-CReSA), 08193 Bellaterra, Barcelona, Spain; 3 Departament de Sanitat i Anatomia Animals, Facultat de Veterinària, Campus de la Universitat Autònoma de Barcelona (UAB), 08193 Bellaterra, Barcelona, Spain

**Keywords:** porcine epidemic diarrhea virus (PEDV), strain, cell passaged, S-INDEL, non-S INDEL, piglet

## Abstract

Porcine epidemic diarrhea virus (PEDV) is characterized by diarrhea, vomiting, dehydration, and high mortality rates in neonatal piglets. Two distinct genogroups, S-INDEL (G1a, G1b) and non-S INDEL (G2a, G2b, and G2c), circulate worldwide and are characterized by varying degrees of virulence. Here, we compared the early pathogenesis of a PEDV S-INDEL strain obtained from intestine homogenate (CALAF-HOMOG) or adapted to cell culture by 22 passages (CALAF-ADAP) and a virulent non-S INDEL strain (PEDV-USA) in newborn piglets. After orogastric inoculation of PEDV strains, body weight, temperature and clinical signs were monitored for 48 hpi. Pathological studies were performed at 48 hpi and RNA extracts from jejunal content (at 48 hpi) and rectal swabs (at 0 and 48 hpi) were tested for the presence of PEDV RNA as well as sequenced and compared to the inoculum. Piglets inoculated with PEDV-USA and CALAF-HOMOG isolates showed more severe weight loss, diarrhea, villi fusion and atrophy compared to CALAF-ADAP inoculated piglets. The viral load of rectal swabs was higher in the PEDV-USA inoculated group, followed by CALAF-HOMOG and CALAF-ADAP isolates. Similarly, viral RNA load in jejunal content was comparable among PEDV-USA and CALAF-HOMOG inoculated piglets and higher than that of CALAF-ADAP ones. The comparison of three full PEDV sequences of the inocula with the corresponding ones of pigs after 48 hpi yielded a nucleotide identity >99.9%. This study highlights variations in virulence among S-INDEL and non-S INDEL strains and between S-INDEL isolates obtained from homogenate and cell culture.

## 1. Introduction

Seven different species of coronaviruses (CoV) have been described in swine to date. Most of them correspond to the genus *Alphacoronavirus*, including porcine epidemic diarrhea (PED) virus (PEDV), transmissible gastroenteritis virus (TGEV), porcine respiratory coronavirus (PRCV), swine enteric coronavirus (SeCoV), and swine acute diarrhea syndrome coronavirus (SADS-CoV). In addition, porcine deltacoronavirus (PDCoV) and porcine hemagglutinating encephalomyelitis virus (PHEV) are the only viruses of the *Deltacoronavirus* and *Betacoronavirus* genera, respectively, that currently affect the porcine species [1]. No aammacoronaviruses have been described in pigs so far.

After its identification in Belgium in 1978, PEDV has been reported in American, European, and Asian pig industries over the last 30 years [2]. PEDV is considered a re-emerging viral species in Europe and Asia and emerging in the USA [3]. The global PEDV paradigm changed in 2010 when highly virulent PEDV strains emerged in China, resulting in devastating outbreaks [4]. Later, from 2013 to 2014, PEDV was reported for the first time in the USA, causing significant economic losses and killing more than 7 million pigs [5,6]. In January 2014, subsequent PED outbreaks with milder clinical signs were also detected in USA farms. Sequence analyses identified in these milder cases corresponded to new PEDV variants characterized by the presence of multiple insertions and deletions on the spike (S) gene; these new strains were called S-INDEL strains [7,8]. S-INDEL strains are responsible for the current outbreaks of PEDV in Europe and in some Asian countries, while the highly pathogenic strains of PEDV (non-S INDEL) continue causing economically important problems in the pig industry of the USA and Asia today [2].

Some porcine coronaviruses (PCoVs) cause severe gastrointestinal disease with almost identical clinical signs characterized by watery diarrhea, dehydration, variable vomiting, cachexia, and even death [9]. The severity of outbreaks depends on several host and viral factors. The most important host-associated risk factors are age and the intestinal innate immune response elicited against PCoVs. The progress of the disease caused by these viruses is characterized by high mortality in neonatal piglets and high morbidity but low mortality in weaned or older pigs [3]. It is generally accepted that the difference in susceptibility and age-dependent pathogenesis responds to immaturity in the development of the gastrointestinal tract and to a poorer intestinal innate immune response in newborn piglets compared with weaned pigs [10,11]. Nonetheless, comparative studies on the innate immune response across different age groups are absent, emphasizing the need for further research to explore its involvement in disease pathogenesis. On the other hand, the viral factor that influences mostly the disease outcome is the ability of the different viral strains (S-INDEL or non-S INDEL) to evade the intestinal type I and III interferon (IFN) antiviral responses. Also, the virus exerts significant effects on the regulation of the main intercellular pathways linked to apoptosis, the endoplasmic stress response and autophagy [12].

The loss of PEDV virulence following its adaptation in Vero cells has been described in previous experimental in vivo and ex vivo studies [3]. Despite the thorough analysis and comparison of the complete genomic sequences of both cell-adapted or field strains, the molecular basis linked to the decreased virulence in the cell-adapted PEDV strains is yet to be fully understood [13]. On the other hand, pathogenesis studies comparing PEDV strains from the USA in conventional neonatal piglets demonstrated that non-S INDEL strains are generally more pathogenic than S-INDEL strains. Specifically, non-S INDEL isolates showed increased severity across all analyzed parameters, including clinical signs, fecal viral shedding, and gross as well as histopathological lesions [13]. However, there is a lack of experimental comparative studies between global and European strains to understand why the pathogenesis and impact of European strains are limited, considering the more severe scenarios of Asia and the USA [6].

Therefore, the aim of the present study was to compare the acute pathogenesis of European S-INDEL and USA non-S INDEL PEDV isolates in a 1-week-old piglet model. Also, we compared the pathogenesis of the same European S-INDEL strain coming from an intestine homogenate of an affected piglet with the corresponding PEDV isolate after several passages in Vero cells. Finally, we discussed the possible molecular bases that might be related to the loss of virulence of the cell-adapted isolate.

## 2. Materials and Methods

### 2.1. Viral Inoculum

Isolation and characterization of the European G1b PEDV S-INDEL strain Calaf 2014 (GenBank accession number MT602520.1) have been described elsewhere [14]. Briefly, the inoculum was obtained from four 3-day-old piglets intragastrically inoculated with 2 mL of the intestinal content of conventional piglets with confirmed PEDV infection and showing diarrhea. Small intestine content diluted 1/100 with 1× phosphate-buffered saline (PBS) solution was obtained at 48 h post-inoculation (hpi) when animals were euthanized after developing severe diarrhea. This suspension, after filtration (Merck & Co., Darmstadt, Germany, SLGV013SL), was used directly as experimental inoculum and named CALAF-HOMOG. On the other hand, the same virus was subsequently isolated, adapted and passaged in Vero Cells (ATCC, CCL-81) with 10 µg/mL of trypsin following a previously described procedure [14]. This inoculum was used in this study and named CALAF-ADAP (viral titer of 10^5.3^ TCID_50_/mL, passage 22).

The isolation and characterization of the USA PEDV isolate USA/NC49469/2013 (GenBank accession number KM975737) have been reported elsewhere [13]. This strain was kindly provided by Dr. J.Q. Zhang (Iowa State University Veterinary Diagnostic Laboratory, USA). Propagation of the virus on Vero cells (ATCC, CCL-81) yielded a virus to a viral titer of 10^6^ TCID_50_/mL (passage 8).

All PEDV inoculum used in this study were confirmed negative for TGEV, porcine rotavirus A (PRV-A) (both pathogens tested with Thermo Fisher Scientific, Carlsbad, CA, USA, 4486975), porcine reproductive and respiratory syndrome virus (PRRSV) (Thermo Fisher Scientific, Carlsbad, CA, USA, A35751) and porcine circovirus 2 (PCV-2) (Thermo Fisher Scientific, Carlsbad, CA, USA, QPCV). For this purpose, inoculum samples were centrifuged at 2500 rpm at room temperature (RT), and RNA and DNA extraction was performed with the MagMAX pathogen RNA/DNA kit (Thermo Fisher Scientific, Carlsbad, CA, USA, 4462359) according to the manufacturer’s instructions.

### 2.2. Sequencing and Phylogenetic Analysis of the Inocula

A comparison of complete genome sequences of the three inocula was carried out previously to the experimental phase. The complete sequence of the CALAF-HOMOG isolate was obtained from the GenBank database (MT602520.1). The complete sequence of the isolates CALAF-ADAP and PEDV-USA was subjected to genome sequencing. In addition, a comparison of the complete genome sequence was also carried out between the Vero cell-passed PEDV-USA inoculum and the original strain obtained from the GenBank database (KM975737). The sequencing procedure was carried out at the Institute of Biotechnology and Biomedicine (IBB) of *the Universitat Autònoma de Barcelona (UAB)* following a previously established protocol. Briefly, sequencing was performed using the Illumina Miseq Platform, following RNA extraction using MagMAX pathogen RNA/DNA kit. RNA purification assessment was performed before sequencing using BioDrop µLite (Isogen Life Science, Utrecht, The Netherlands, 80-3006-51). The translation of the nucleotide to amino acid sequence was obtained with the Expasy program and the alignment of the nucleotide and amino acid sequences with the Clustal Omega command included in the unified bioinformatics toolkit Unipro UGENE [15]. The graphics were obtained using the program Geneious Prime by Dotmatics (version 2023.1.2).

A phylogenetic analysis was carried out with PEDV sequences from inocula, which were aligned together with a total of 53 PEDV complete genome sequences available in the GenBank database using the Clustal Omega v1.2.4 software. The MEGA-X software was employed to determine the optimal substitution method, and the Seaview v5.0.5 software was utilized to validate and rectify the alignment as well as to construct the phylogenetic tree. Maximum Likelihood was used as the reconstruction method, and the branch supports were calculated by using the Shimodaira–Hasegawa (SH) approximate likelihood ratio test (aLRT) [16].

### 2.3. Experimental Design

A total of twenty 3–5-day-old piglets from 5 sows, negative by RT-PCR for PEDV and TGEV in feces and with no antibodies against both viruses, were selected and transported to the experimental farm (IRTA-Monells, Girona, Spain). Sows were also negative against PRRSV and PCV-2 in serum by RT-qPCR and qPCR, respectively. Upon arrival at the facilities, animals were weighed and randomly distributed into four pens of 5 animals/pen based on body weight, gender, and sow origin. Four experimental groups were considered attending to the inoculum administered: (1) S-INDEL PEDV strain Calaf 2014 passaged in Vero cells (CALAF-ADAP), (2) S-INDEL PEDV strain Calaf 2014 from intestine homogenate (CALAF-HOMOG), (3) non-S INDEL PEDV strain USA NC4969/2013 passaged in Vero cells (PEDV-USA), and (4) PBS (negative control group, C-NEG).

At study day (SD) 0, all animals were orogastrically inoculated with 5 × 10^4.5^ TCID_50_ of each of the corresponding PEDV isolates in a 5 mL volume or with saline solution (C-NEG).

Pigs were scored for clinical signs (rectal temperature and diarrhea) at 0 hpi before challenge and every 12 h until necropsy (48 hpi). Diarrhea severity was assessed using a scoring system: 0, normal feces; 1, soft and/or pasty feces; 2, liquid feces with some solid content; and 3, watery fecal content. A rectal temperature below 40 °C was considered normal and ≥40 °C was considered fever.

Body weight and blood samples were recorded and collected at 0 hpi (before challenge) and repeated at necropsy (48 hpi). Rectal swabs were collected before challenge (0 hpi) and at 24 and 48 hpi in 500 µL of Minimum Essential Media, MEM (Thermo Fisher Scientific, Carlsbad, CA, USA, 21090022) supplemented with 1% penicillin/streptomycin (Thermo Fisher Scientific, Carlsbad, CA, USA, 15140122) to determine fecal PEDV shedding by RT-qPCR and virus isolation. Viral infectivity (expressed as log10 TCID_50_/mL) was extrapolated from the Ct values of the jejunum content using a standard curve, as previously described [17].

### 2.4. Nucleic Acid Extraction and RT-PCR/PCR for Detection of Viral Pathogens

Viral RNA extraction from feces and swabs at 0, 24 and 48 hpi, and from small intestinal (jejunum) content at 48 hpi were done following the procedure previously described [14]. Briefly, rectal swabs and feces were diluted with PBS supplemented with 1% penicillin/streptomycin (feces were diluted 1/10), and after vortexing the samples, RNA extraction was performed with the MagMAX pathogen RNA/DNA kit described previously according to the manufacturer’s instructions. Detection and quantification of PEDV, TGEV and Rotavirus A (PRV-A) were performed with the commercial qPCR kits described previously. Also, PRRSV and PCV-2 were ruled out in serum from all pigs at 0 hpi by means of the previously mentioned RT-qPCR and qPCR methods, respectively. Results were expressed as Ct values as the expression of the viral load in serum and/or resuspended feces/swabs.

### 2.5. ELISA Tests

The presence of antibodies against PEDV and TEGV in serum at 0 hpi was tested using commercial ELISAs Ingezim PEDV (Ingenasa, Madrid, Spain, 11.PED.K1) and Ingezim TGEV 2.0 (Ingenasa, Madrid, Spain, 11.TGE.K3) kits, respectively, according to the manufacturer’s instructions. Results were expressed as mean S/P titers. For PEDV, they are categorized as either positive (>0.35) or negative (<0.35), while for TGEV, they are classified as positive (<1.0008), negative (>1.1676), or doubtful (1.0008–1.1676).

### 2.6. PEDV Genome Sequencing

Jejunal contents at 48 hpi of all experimentally inoculated animals were subjected to viral sequencing at the Institute of Biotechnology and Biomedicine (IBB) of *the Universitat Autònoma de Barcelona (UAB)* following the procedure described above (Section 2.2). One animal from each group (the one with the lowest Ct value) was selected to compare the complete genomic sequences between the different PEDV strains recovered at 48 hpi with the corresponding inoculum.

### 2.7. Pathological Analyses and Immunohistochemistry

All pigs were euthanized at 48 hpi with an overdose of pentobarbital followed by exsanguination. Complete necropsies were performed, and the small intestine (jejunum and ileum), caecum, colon and mesenteric lymphnode (LN) were examined for gross lesions by ECVP-certified veterinary pathologists, blinded to the treatment groups. Intestinal wall findings were scored as: 0, normal; 1, thin-walled or gas-distended; 2, presence of both. Contents of the small intestine, caecum and colon were examined and scored following the scoring system: 0, normal; 1, pasty; or 2, watery content.

Small intestine, colon and mesenteric LN were collected in 10% neutral-buffered formalin and embedded in paraffin. Tissues were processed and stained with hematoxylin and eosin (H&E) using standard laboratory procedures. Villus lengths and crypt depths were measured from ten representative villi and crypts of middle jejunum per animal [18]. The Villus-height-to-crypt-depth (VH:CD ratio) ratio was calculated as the quotient of the mean villus length divided by the mean crypt depth.

An immunohistochemistry (IHC) technique to detect PEDV nucleoprotein using a rabbit monoclonal antibody (Medgene Labs, Brookings, SD, USA, SD6-29) at dilution 1/5000 was performed on selected tissues (jejunum, ileum, colon and mesenteric lymph node). After deparaffinization and hydration of the samples, peroxidase blocking was performed with methanol 3% H_2_O_2_ for 30 min at RT. Antigen retrieval with citrate buffer pH6 (Dako/Agilent Technologies, Santa Clara, CA, USA, S169984-2) at 98 °C for 20 min plus 30 min at RT was performed after washing the samples with PBS. The primary antibody was added after blocking the specific binding with PBS-Tween 20% and 2% Bovine Serum Albumin (BSA). After overnight incubation with the primary antibody at 4 °C, the CRF-Anti-polyvalent HRP Polymer/ScyTeck (Quimigen, Madrid, Spain, ABZ125) and 3,3′-diaminobenzidine (DAB) (Sigma-Aldrich/Merck & Co, Darmstadt, Germany, D5637) were added as secondary antibody and chromogen substrate, respectively. Finally, slides were counterstained with hematoxylin.

For intestinal tissues, the amount of viral antigen was semiquantitatively scored from 0 to 3. Score 0 was assigned to tissues with no PEDV staining. Scores 1, 2, and 3 were assigned to tissues with less than 30%, 30–60%, or more than 60% of PEDV-immunolabeled enterocytes, respectively. The evaluation was performed in 10 high-power fields (HPF; magnification, 400×). The IHC scoring in mesenteric LN, also from 0 to 3, was assigned to tissue with a lack of immunolabeled cells (0), and the detection of 10–30, 30–50 and more than 50 PEDV-immunolabeled cells received scorings of 1, 2 and 3, respectively.

### 2.8. Statistical Analyses

Comparisons between groups and times of body weight, temperature and VH:CD ratio were calculated using ANOVA. The analysis of diarrhea, gross findings and histopathological data was performed using a generalized linear model with a binary response. The Ct values of the PEDV RT-qPCR of the rectal swabs and jejunum contents were compared among groups using the Kruskal–Wallis Test. In all tests, *p*-values for pairwise comparisons were adjusted for multiplicity using Tukey’s correction for parametric tests and Bonferroni’s correction for nonparametric tests. Statistical analyses were performed with the SAS system version 9.4 (SAS Institute Inc., Cary, NC, USA). Graphics were generated using GraphPad Prism 8. In all analyses, a *p*-value < 0.05 was considered statistically significant.

## 3. Results

### 3.1. Both CALAF Inocula Had Similar Genomes and Showed More Variation in Their Sequences When Compared to the PEDV-USA One

The CALAF-ADAP inoculum showed a high nucleotide identity compared to CALAF-HOMOG, displaying a consistent 99.91% similarity between their sequences. Likewise, the PEDV-USA strains (original and cell-passaged) exhibited 99.98% similarity. In contrast, the CALAF-HOMOG and cell-passaged PEDV-USA genomes were only 98.7% identical, revealing 356 discrepancies in their nucleotide sequences. These variations were notably concentrated within the amino acid sequence of the S protein, followed by the ORF1ab, N, ORF3b, and M viral proteins across all PEDV inocula. The amino acid sequence differences between both CALAF inocula were primarily concentrated in Nsp-2, -3 (PLPro), -4, -12 (RdRp), and -15 (NendoU), within the NTD/SO and RBD sequences of S1, and within Heptad Repeat (HR) 1 and 2 of S2. Remarkably, this investigation unveiled the exceptional conservation of the envelope protein across various PEDV strains, with no alterations observed even after extensive cell-culture passaging.

The complete genomes of the CALAF-HOMOG and PEDV USA strains were compared to a total of 53 global PEDV strains sourced from GenBank (Supplementary Table S1). Our results confirmed that both PEDV strains belong to distinct genogroups, specifically, S-INDEL and non-S INDEL, respectively.

The phylogenetic analysis revealed that European PEDV strains re-emerging since 2010 belong to the S-INDEL genogroup, primarily within the G1b clade, except for the 2014 Ukraine strain. This latter strain clusters within the G2b clade alongside non-S INDEL strains from America and Asia, consistent with prior findings by Dastjerdi et al. [19]. The close alignment of the Calaf 2014 strain with most European strains from 2014–2015 implies a common origin for PEDV reintroduction into Europe in 2014. Notably, American and Asian S-INDEL strains within the G1b clade (Indiana12.83/2013, Strain OH851/2014, Minnesota58/2013, and SK/KNU-1406/2014) show relative proximity to European S-INDEL strains from 2014–2015, suggesting potential origins for re-emerging PEDV strains in Europe as proposed by Hanke et al. [20,21]. Conversely, the non-S INDEL USA strain (NC/2013/49469) closely aligns with other American PEDV strains from 2013–2014, all falling within the G2a clade, indicating widespread dissemination of PEDV in the United States since its introduction in 2013 (Supplementary Figure S1).

### 3.2. Clinical Outcome Varies Depending on the PEDV Isolate

**Figure 1:**
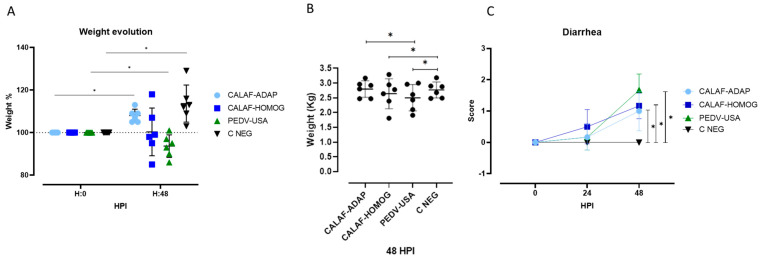
(**A**) Individual and average weight (represented in % with respect to the initial weight) of the experimental groups at 0 and 48 hpi. While piglets inoculated with the PEDV-USA strain showed weight loss at 48 hpi, piglets inoculated with CALAF-ADAP and those from C-NEG gained weight (*p* < 0.05). No significant differences in weight were observed in the CALAF-HOMOG group between 0–48 hpi. (**B**) Individual (dots) and average (line) weight (in kg) of studied piglets within each experimental group at 48 hpi. The PEDV-USA strain caused a significant weight reduction compared to the rest of the groups, but no significant differences were observed between the different study groups. (**C**) Evolution of diarrhea from all experimental groups until the end of the study (48 hpi). Score for diarrhea: 0 = Normal stools; 1 = Soft and/or pasty feces; 2 = Liquid with some solid content and 3 = Watery feces. Pasty diarrhea started at 24 hpi in both the PEDV-USA and CALAF-HOMOG groups, but the CALAF-ADAP strain caused mild (pasty) diarrhea later than the other groups (48 hpi). At 48 hpi, the PEDV-USA group showed severe watery diarrhea compared to the CALAF-HOMOG group. No differences were observed between the CALAF-HOMOG and CALAF-ADAP groups at the end of the study. Asterisk means significant difference between groups (*p* < 0.05).

Compared to starting weight (0 hpi), PEDV-USA-inoculated piglets showed a significant weight loss (*p* < 0.05) at 48 hpi, while no major weight variation was observed in CALAF-HOMOG piglets, and animals from PEDV-ADAP and C-NEG groups significantly gained weight (*p* < 0.05) (Figure 1A). Weight at 48 hpi was significantly different among groups, with the lower weight for the PEDV-USA inoculated piglets followed by the CALAF-HOMOG ones and the remaining two groups; no significantly different weight at 48 hpi was observed between piglets that received CALAF-ADAP or PBS (Figure 1B).

All three groups inoculated with PEDV isolates developed diarrhea (Figure 1C), which varied between average scores 1 and 2. Diarrhea was first detected at 24 hpi with a score of 1 (pasty diarrhea) in both the PEDV-USA and CALAF-HOMOG groups and evolved to liquid (score of 2) only in the PEDV-USA group at 48 hpi. Regarding PEDV-ADAP, diarrheas had a slower and milder course, characterized by soft feces (score of 1) at 48 hpi. Even so, no significant differences were observed between the PEDV-USA, CALAF-HOMOG and CALAF-ADAP groups in terms of mean diarrhea scoring (*p* < 0.05). No vomiting or fever was observed in any piglet throughout the study.

### 3.3. PEDV Antigen Was Present in the Jejunum and Ileum, but Only the PEDV-USA and CALAF-HOMOG Isolates Caused Intestinal Damage

**Figure 2:**
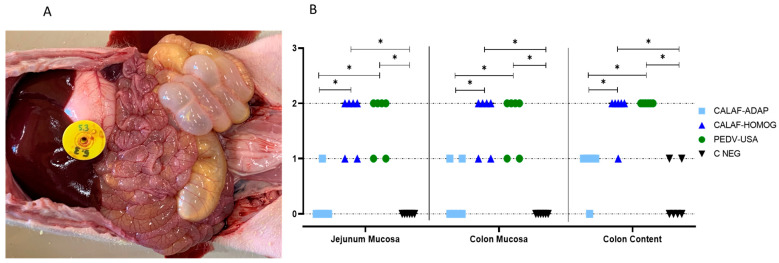
Gross pathological findings at 48 hpi after inoculation of the PEDV-USA strain. (**A**) Severe form of the disease characterized by marked dilation of the intestinal loops with large amounts of gas and fluid content in both the small and large intestines. (**B**) Damage to the mucosa (wall) of the jejunum and colon at 48 hpi (ranging from normal (0) to thin-walled and gas-distended wall (2)) and content of the colon at 48 hpi (solid (0); pasty (1) and watery (2) content). Both PEDV-USA and CALAF-HOMOG strains caused similar severe wall lesions in the jejunum and colon at 48 hpi, characterized by thin and gas distended-walls. However, no significant lesions were observed in the jejunum and colon wall in CALAF-ADAP and negative control group. Furthermore, both PEDV strains (PEDV-USA and CALAF-HOMOG) caused severe watery diarrhea in the large intestine at 48 hpi compared to the CALAF-ADAP strain, which caused pasty diarrhea (*p* < 0.05). Asterisk means significant difference between groups (*p* < 0.05).

The summary of individual scores related to intestinal wall and digestive contents is displayed in Figure 2. At 48 hpi, both the PEDV-USA and CALAF-HOMOG strains caused similar lesions in the enteric tract regarding the studied parameters (Figure 2A); interestingly, the intestinal wall and content scores caused by the PEDV-ADAP were significantly lower (*p* < 0.05). In the PEDV-USA and CALAF-HOMOG groups, the intestinal wall was thin and transparent, distended by gas (score 2) and with yellowish watery contents (score 2) (Figure 2B). On the other hand, the CALAF-ADAP strain caused no or mild lesions in the intestinal wall characterized by mildly thin or gas-distended walls (score 1) with pasty contents (score 1), which were similar to the C-NEG group.

**Figure 3:**
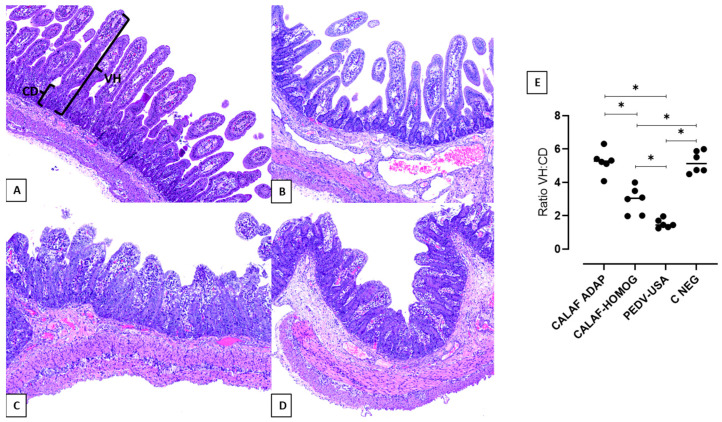
Jejunum at 48 hpi from the negative control (**A**), CALAF-ADAP (**B**), CALAF-HOMOG (**C**) and PEDV USA (**D**) stained with hematoxylin and eosin (H&E). While no histological damage was observed in groups A and B, similar severe villous atrophy and fusion were observed in both C and D groups. (**E**) The degree of intestinal damage was measured by the villus height/crypt depth ratio (VH:CD). PEDV-USA caused a higher degree of atrophy and fusion of villi at 48 hpi, followed by PEDV-HOMOG. However, PEDV-ADAP did not cause epithelial damage (*p* < 0.05). Asterisk means significant difference between groups (*p* < 0.05).

Histological intestinal lesions in PEDV inoculated piglets consisted of enterocyte swelling and villous fusion and atrophy (Figure 3A–D) and were severe in jejunum and ileum in all piglets from PEDV-USA and CALAF-HOMOG groups at 48 hpi. In addition, atrophic enterocytes of these experimental groups showed cytoplasmic vacuolation (enterocyte degeneration), cell hypereosinophilia, cell retraction and nuclear pyknosis (enterocyte necrosis) and epithelial attenuation in the apical villi (Supplementary Figure S2A). None of these features were observed in small intestine sections of animals inoculated with the CALAF-ADAP isolate or in piglets that received PBS.

Intestinal atrophy and fusion were characterized by a marked reduction in intestinal VH, causing a decreased VH:CD ratio (Figure 3E), which was significantly (*p* < 0.05) more severe in the PEDV-USA-inoculated pigs compared to the ones of the CALAF-HOMOG group (mean VH:CD ratio of 1.52 and 3, respectively). No intestinal lesions were observed in piglets from the PEDV-ADAP group, which showed a similar VH:CD ratio than C-NEG animals (mean VH:CD ratio of 5.5) (Figure 3E).

**Figure 4:**
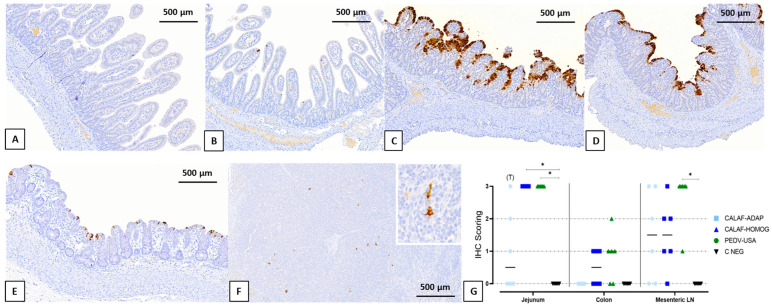
Detection of PEDV by immunohistochemistry (IHC) in sections of jejunum at 48 hpi from the negative control (**A**), CALAF-ADAP (**B**), CALAF-HOMOG (**C**) and PEDV-USA (**D**). Scattered PEDV-immunolabeled apical enterocytes were observed in the PEDV-ADAP group, whereas it was detected as widespread in the cytoplasm of enterocytes from the apical part of atrophic and fused villi in both PEDV-HOMOG and PEDV-USA. Scattered PEDV-immunolabeled apical enterocytes were also observed in a few colonic apical enterocytes (**E**) and in dendritic-like cells of the MLN (**F**) from a piglet inoculated with PEDV-USA strain. (**G**) Detection of PEDV by IHC in the middle jejunum, colon, and mesenteric lymph node at 48 hpi. PEDV was mainly detected in the middle jejunum in a similar distribution both for the PEDV USA and PEDV-HOMOG strains and, less frequently, in the colon. PEDV-ADAP was less observed in the middle jejunum compared with the other strains. PEDV labeled was observed for the three viral strains in the MLN at 48 hpi, but it was more frequent and constant in the PEDV USA group. Asterisk means significant difference between groups (*p* < 0.05).

PEDV antigen showed diffuse and granular staining patterns and was mainly located in the cytoplasm of the enterocytes of the apical part of the villi of the jejunum (Figure 4A–D) and ileum. While few PEDV-immunolabeled apical enterocytes were observed in the CALAF-ADAP-inoculated pigs, it was detected widespread in the cytoplasm of enterocytes in both PEDV-HOMOG and PEDV-USA piglets, coinciding with the areas of atrophy and fusion of villi. Occasionally, viral antigen was also observed in the cytoplasm of epithelial cells of the crypts of Lieberkühn and in interstitial mononuclear cells (compatible with histiocytic cells or dendritic cells) widespread throughout the lamina propria (Supplementary Figure S2B and Figure S2C, respectively). PEDV antigen was also observed in few colonic apical enterocytes only in the case of PEDV-USA and CALAF-HOMOG inoculated piglets and in dendritic-like cells of the mesenteric LN in all PEDV inoculated animals (Figure 4E and Figure 4F, respectively). Although differences in viral detection by IHC were observed in the small and large intestines and in mesenteric lymph nodes among the three PEDV strains, these were not statistically significant (Figure 4G). Nevertheless, a notable trend emerged when comparing the CALAF-ADAP strain with the other two strains in the jejunum sections (*p* < 0.05). Furthermore, the PEDV antigen detection in the mesenteric LN was greater in the PEDV-USA strain when contrasted with both CALAF strains in terms of mean IHC scoring (mean of 3 in PEDV-USA group and 1.5 in both CALAF groups).

### 3.4. All PEDV Isolates Replicated in the Jejunum and Were Shed in Feces at 48 hpi with Different Viral Loads Depending on the Isolate

The detection of PEDV RNA was analyzed by RT-qPCR in rectal swabs (0 and 48 hpi) and jejunal content (48 hpi) from all piglets. Viral inoculum, as well as all fecal samples throughout the whole study, were negative for TGEV and PRV-A, as determined by RT-qPCR.

**Figure 5:**
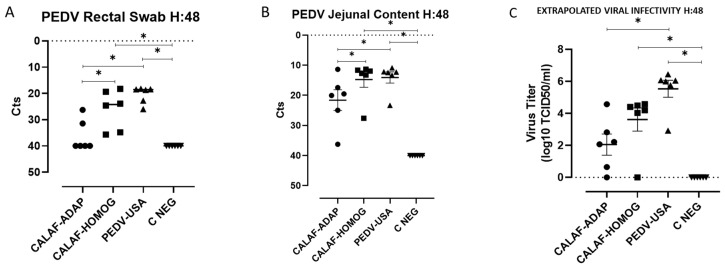
PEDV loads detectable in rectal swabs (**A**) and jejunal content (**B**) at 48 hpi. All PEDV isolates replicated in jejunal segments and were shed in faces at 48 hpi. No significant differences in viral load were observed between the CALAF HOMOG and PEDV-USA groups, but both were significantly higher than the CALAF-ADAP group. Estimated viral titer (**C**) (expressed as log10 TCID_50_/_mL_) from jejunal content Ct values at 48 hpi. PEDV USA was the strain with the highest infectious virus titer, followed by the PEDV-HOMOG and PEDV-ADAP strains. Asterisk means significant difference between groups (*p* < 0.05).

At 0 hpi, rectal swabs from all groups were negative for PEDV. However, the virus was detected in rectal swabs (Figure 5A) and jejunal contents (Figure 5B) at 48 hpi in all three viral inoculated groups, indicating that all PEDV isolates replicated and were shed in feces at that time postinoculation. Although no significant differences were observed between the viral load of intestinal content or rectal swabs between the PEDV-USA and CALAF-HOMOG groups, their amounts were significantly higher than that of the CALAF-ADAP group (*p* < 0.05). No PEDV RNA was detected in any C-NEG piglets.

Viral titers from jejunum content (extrapolated from Ct values) indicated that those of PEDV-USA strain were the highest ones, followed by the CALAF-HOMOG and the CALAF-ADAP isolates (viral titers of around 6, 4 and 2 log10 TCID_50_/mL, respectively) (Figure 5C). Significant differences in viral titers among inoculated groups were only observed between the PEDV-USA and CALAF-ADAP strains (*p* < 0.05).

### 3.5. Sequences of Inocula and Viruses Recovered from Intestinal Contents at 48 hpi Were Highly Conserved

After 48 hpi, the PEDV sequences retrieved from the jejunal contents from inoculated piglets had a high degree of genomic similarity nucleotide identity with the consensus sequences of the PEDV-ADAP, CALAF-HOMOG, and PEDV-USA inocula (100%, 99.91%, and 99.98%, respectively).

## 4. Discussion

The present work focused on elucidating the early pathogenesis and severity of the clinical disease in a newborn piglet model for PEDV using different isolates. Specifically, we compared the clinical, pathological and virological outcomes of cell-passaged USA non-S INDEL (PEDV-USA) and European S-INDEL (CALAF-HOMOG), an intestine homogenate from a piglet inoculated with intestinal content of piglets infected with PEDV, as well as its cell culture adapted isolate (CALAF-ADAP).

Upon comparing the sequences of different inocula, notable distinctions among the three PEDV isolates were observed in the sequences of the nonstructural (Nsp) and S proteins, followed by N, M and ORF3b proteins. These viral proteins have been previously demonstrated to counteract host antiviral mechanisms through diverse molecular pathways, enhancing the virus’s ability to evade innate immunity and promoting viral replication [22,23,24,25]. The role of these viral proteins in the clinical, pathological and virological differences between PEDV S-INDEL and non-S INDEL strains requires comprehensive studies to further investigate this hypothesis. The sequencing analysis of fecal samples of inoculated piglets revealed the preservation of the PEDV genome at 48 hpi, remaining almost identical to the initial inoculum across all three PEDV isolates. This implies that viral variation within the first 48 hpi is limited; the short period of time is probably not enough to accumulate significant mutations.

Globally, the results obtained in this study aligned with previous studies [3,13], demonstrating two major issues. First, the adaptation of PEDV in Vero cells (i.e., CALAF-ADAP isolate after 22 cell culture passages) attenuated the virulence of the original strain. In this study, the cell-adapted S-INDEL isolate did not reproduce evident clinical signs or cause intestinal lesions when compared to the same strain derived from intestinal homogenate. Notably, the differences observed between the genomic sequences of different PEDV inocula could have arisen subsequently to cell passages, suggesting an important role in the attenuation of the cell-adapted PEDV strain. The differences between the two S-INDELs inocula genomes in this study were exclusively located in Nsp2, 3 (PLPro), 4, 12 (RdRp) and 15 (NendoU) genes, within the ORF1a/b, as well as receptor binding domain (RBD), HR1 and HR2 within the S gene. In many coronaviruses and other RNA viruses, HR1- and HR2-coded proteins progressively interact to form a six-helix bundle (6-HB), facilitating viral entry into host cells and resulting in complete fusion between the viral envelope and the host cell membrane [23,26]. These genomic differences suggested that the attenuation of cell-adapted PEDV strains after several cell passages may be related to the synthesis of IFN antagonists (regulated by Nsp) or by preventing the fusion and entry of the virus into the cell (either due to a receptor alteration or an abnormal formation of the 6-HB).

A second issue of the present study was the fact that the cell-passaged non-S INDEL isolate (PEDV-USA) caused more severe disease in newborn piglets compared to the cell-passaged S-INDEL one (CALAF-ADAP). However, the tissue homogenate derived from a PEDV S-INDEL (CALAF-HOMOG) exerted comparable effects to the cell-passaged non-S INDEL for most of the studied parameters. Virulence attenuation has also been reported in non-S INDEL strains after multiple cell passages in Vero cells [13,27,28,29]. The non-S INDEL isolate used in this experiment was previously passed by CCL-81 Vero cells prior to the challenge (passage 8). Considering that cell-passaging tends to attenuate PEDV isolates [3], it is very likely that a putative non-S INDEL tissue homogenate would have caused an even more severe outcome compared to the CALAF-HOMOG strain used in the present study. Noteworthy, the potential virulence attenuation by cell passaging of the non-S INDEL isolate was probably lower than the S-INDEL one since the former one was subjected to eight passages while the latter one to 22. The obtained results further support a previous study showing that the adaptability and loss of virulence of PEDV to cells increases with the number of cell passages [29].

In fact, the higher virulence of non-S INDEL compared to S-INDEL isolates has been widely demonstrated [3,13]. Consistent with these investigations, the results of our comparative study indicate that non-S INDEL strains induced more pronounced intestinal villi atrophy and fusion in the jejunum and ileum segments compared to S-INDEL strains. In general terms, the greater the degree of intestinal atrophy, the smaller the absorptive surface, causing greater malabsorptive diarrhea [30]. Accordingly, the loss of pathogenicity of the cell-adapted S-INDEL strain was linked to the lack of atrophy and fusion of the jejunal sections.

Noteworthy, non-S INDEL (PEDV-USA) and S-INDEL (CALAF-HOMOG) antigens were located in the jejunum and ileum segments as previously reported during the initial (up to 24 hpi) stages of PEDV infection [1,3,13]. However, the non-S INDEL had a greater ability to spread to the colon and mesenteric LN than the S-INDEL CALAF-HOMOG strain by 48 hpi. The presence of viral antigen in the jejunum coincided with the areas of atrophy and fusion of villi, demonstrating that it is a direct viral-induced damage [31]. Interestingly, the amount of viral antigen was similar in both PEDV-USA and CALAF-HOMOG strains regardless of the degree of atrophy and villous fusion since there were significant differences in VH:CD between both groups. These results could suggest that the degree of enterocyte damage is not strictly related to the amount of virus but depends on other factors. It has been recently demonstrated that different PCoV species, including PEDV, can modulate apoptosis, endoplasmic reticulum stress response and autophagy to favor a viral environment [12]. In the present study, both the PEDV-USA and CALAF-HOMOG strains showed evidence of enterocyte degeneration and death coinciding with areas of villous atrophy and fusion and the presence of viral antigen. These results could suggest that both PEDV strains could induce programmed cell death of PEDV-infected enterocytes to evade the innate immune response. Even so, more precise pathophysiology studies are needed to evaluate the differences in PEDV-associated cell damage between non-S INDEL and S-INDEL strains. On the other hand, PEDV-ADAP antigen was also identified in the small intestine sections, even in the absence of intestinal damage, as observed in previously reported cases with cell-adapted strains [13]. Interestingly, the IHC results of the cell-adapted S-INDEL strain showed an increased ability of this strain to infect segments of the ileum rather than the middle jejunum, which differs from what has been previously described [3].

In addition, there was discernible variation in PEDV antigen detection within the mesenteric LN between non-S INDEL and S-INDEL strains. These findings could imply a potential connection between the disparities observed in both strains and the potential development of an immunological response, possibly influenced by the quantity of PEDV-immunolabeled dendritic-like cells, which could play a significant role in disease response and control. Nevertheless, more extensive studies are needed to validate this hypothesis.

The virological assessment demonstrated that non-S INDEL (PEDV-USA) and S-INDEL (CALAF-HOMOG) strains had similar loads in the jejunum and that they were shed to the equivalent amount in feces at 48 hpi. Since viral load titration was estimated from the RT-qPCR Ct values from intestine contents, these values may not be accurate enough, so it was not possible to definitively assess if the replication capabilities of the two abovementioned strains were truly different. However, considering the amounts of antigen (PEDV nucleoprotein) detected by immunohistochemistry were fairly similar between groups, it is very likely that replication levels were similar among both. On the other hand, the CALAF-ADAP strain had significantly higher Ct values by RT-qPCR compared to the other two isolates, indicating lower loads in the jejunum and feces. This finding aligns with prior research that suggested cell-adapted S-INDEL strains exhibited reduced shedding and replication efficiency compared to the non-cell-adapted S-INDEL strains [3].

## 5. Conclusions

In conclusion, the USA non-S INDEL cell-passaged isolate resulted in a disease outcome similar to that of the European S-INDEL strain from tissue homogenate. However, both of these isolates displayed significant distinctions in clinical, pathological, and virological aspects when compared to the European S-INDEL cell-passaged isolate.

## Data Availability

Original data files are available on request.

## Supplementary Materials

The following supporting information can be downloaded at: https://www.mdpi.com/article/10.3390/v16010044/s1, Figure S1: Phylogenetic analysis of PEDV strains in Europe and the United States; Figure S2: Evidence of PEDV-induced enterocyte damage and the presence of virus (IHC) in jejunal crypts and dendritic-like cells at 48 hpi; Table S1: References of the selected global PEDV sequences from Genbank [32,33,34,35,36,37].
