# Supplementary material for: Clinical, Pathological and Virological Outcomes of Tissue-Homogenate-Derived and Cell-Adapted Strains of Porcine Epidemic Diarrhea Virus (PEDV) in a Neonatal Pig Model
Source: Viruses. 2023 Dec 27;16(1):44. doi: 10.3390/v16010044 (PMC10819582; doi:10.3390/v16010044)
Supplement: Supplementary file 1 [file viruses-16-00044-s001.zip › supplementary figure S1.pdf]

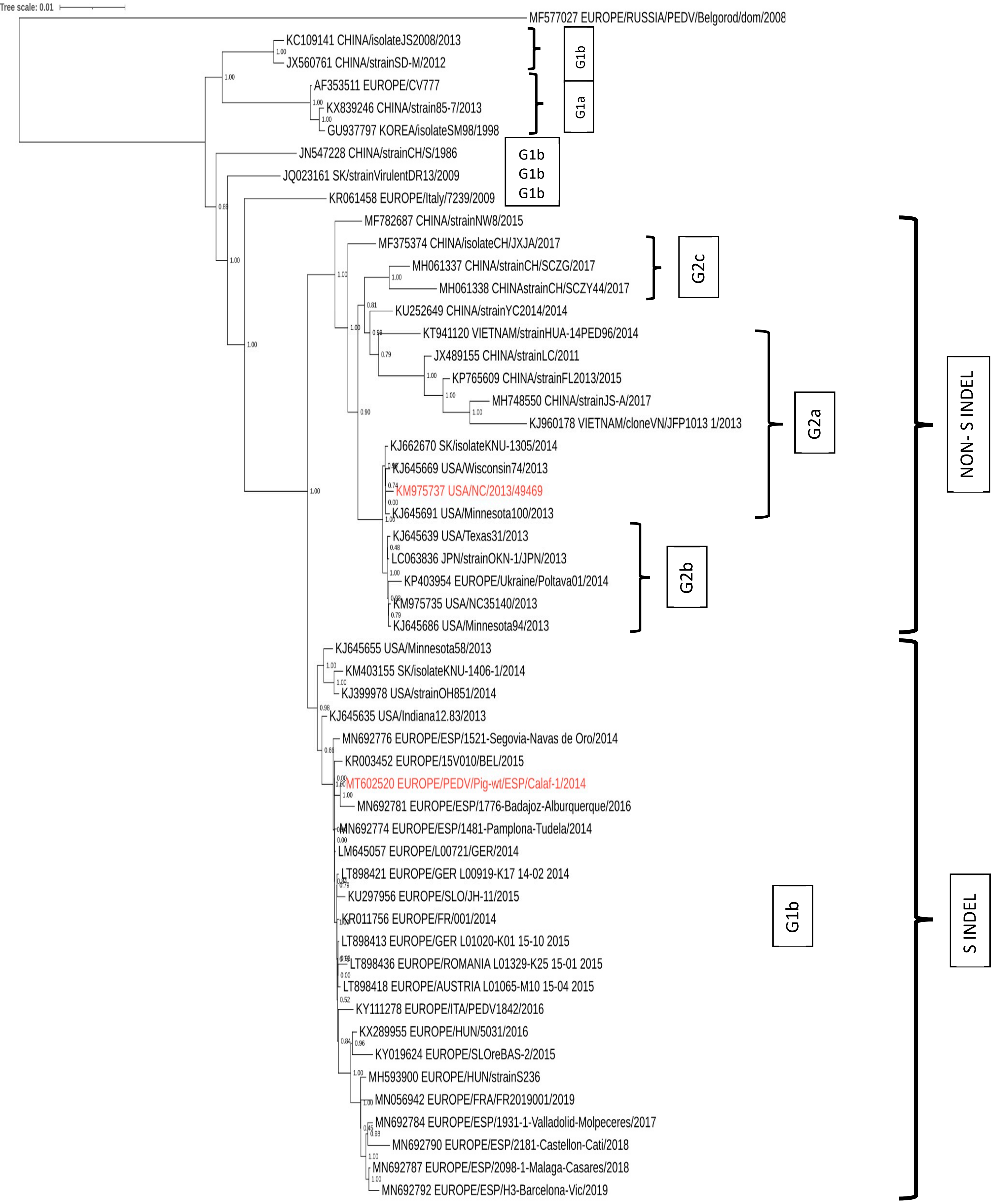

Tree scale: 0.01
MF577027 EUROPE/RUSSIA/PEDV/Belgorod/dom/2008
KC109141 CHINA/isolateJS2008/2013
JX560761 CHINA/strainSD-M/2012
G1b
AF353511 EUROPE/CV777
KX839246 CHINA/strain85-7/2013
GU937797 KOREA/isolateSM98/1998
G1a
JN547228 CHINA/strainCH/S/1986
JQ023161 SK/strainVirulentDR13/2009
KR061458 EUROPE/Italy/7239/2009
G1b
G1b
G1b
MF782687 CHINA/strainNW8/2015
MF375374 CHINA/isolateCH/JXJA/2017
MH061337 CHINA/strainCH/SCZG/2017
MH061338 CHINAstrainCH/SCZY44/2017
G2c
KU252649 CHINA/strainYC2014/2014
KT941120 VIETNAM/strainHUA-14PED96/2014
JX489155 CHINA/strainLC/2011
KP765609 CHINA/strainFL2013/2015
MH748550 CHINA/strainJS-A/2017
KJ960178 VIETNAM/cloneVN/JFP1013 1/2013
KJ662670 SK/isolateKNU-1305/2014
KJ645669 USA/Wisconsin74/2013
KM975737 USA/NC/2013/49469
KJ645691 USA/Minnesota100/2013
G2a
KJ645639 USA/Texas31/2013
LC063836 JPN/strainOKN-1/JPN/2013
KP403954 EUROPE/Ukraine/Poltava01/2014
KM975735 USA/NC35140/2013
KJ645686 USA/Minnesota94/2013
G2b
NON- S INDEL
KJ645655 USA/Minnesota58/2013
KM403155 SK/isolateKNU-1406-1/2014
KJ399978 USA/strainOH851/2014
KJ645635 USA/Indiana12.83/2013
MN692776 EUROPE/ESP/1521-Segovia-Navas de Oro/2014
KR003452 EUROPE/15V010/BEL/2015
MT602520 EUROPE/PEDV/Pig-wt/ESP/Calaf-1/2014
MN692781 EUROPE/ESP/1776-Badajoz-Alburquerque/2016
MN692774 EUROPE/ESP/1481-Pamplona-Tudela/2014
LM645057 EUROPE/L00721/GER/2014
LT898421 EUROPE/GER L00919-K17 14-02 2014
KU297956 EUROPE/SLO/JH-11/2015
KR011756 EUROPE/FR/001/2014
LT898413 EUROPE/GER L01020-K01 15-10 2015
LT898436 EUROPE/ROMANIA L01329-K25 15-01 2015
LT898418 EUROPE/AUSTRIA L01065-M10 15-04 2015
KY111278 EUROPE/ITA/PEDV1842/2016
KX289955 EUROPE/HUN/5031/2016
KY019624 EUROPE/SLOreBAS-2/2015
MH593900 EUROPE/HUN/strainS236
MN056942 EUROPE/FRA/FR2019001/2019
MN692784 EUROPE/ESP/1931-1-Valladolid-Molpeceres/2017
MN692790 EUROPE/ESP/2181-Castellon-Cati/2018
MN692787 EUROPE/ESP/2098-1-Malaga-Casares/2018
MN692792 EUROPE/ESP/H3-Barcelona-Vic/2019
G1b
S INDEL

Supplementary Figure S1. Phylogenetic analysis of PEDV strains in Europe and the United States. (a) European PEDV strains from 2010 onwards belong to the S INDEL genogroup and are primarily part of the G1b clade, except for the 2014 Ukraine strain, which falls under the G2b clade. (b) The Calaf 2014 strain in Europe is genetically close to other European strains from 2014-2015, suggesting a shared origin for PEDV's reintroduction into Europe in 2014. (c) Indiana12.83/2013, Strain OH851/2014, Minnesota58/2013, and SK/KNU-1406/2014 show relative proximity to European S-INDEL strains from 2014-15, suggesting potential origins for re-emerging PEDV strains in Europe. (d) Spanish S INDEL strains exhibit distinct subgroups within separate branches, with strains from 2013-2015 displaying a more branched pattern and strains from 2016-2019 showing high sequence conservation. (e) The NON-S INDEL USA strain (NC/2013/49469) closely aligns with other American PEDV strains from 2013-2014, all within the G2a clade, indicating widespread dissemination of PEDV in the United States since its introduction in 2013.
