# Supplementary material for: Clinical, Pathological and Virological Outcomes of Tissue-Homogenate-Derived and Cell-Adapted Strains of Porcine Epidemic Diarrhea Virus (PEDV) in a Neonatal Pig Model
Source: Viruses. 2023 Dec 27;16(1):44. doi: 10.3390/v16010044 (PMC10819582; doi:10.3390/v16010044)
Supplement: Supplementary file 1 [file viruses-16-00044-s001.zip › supplementary figure S2.pdf]

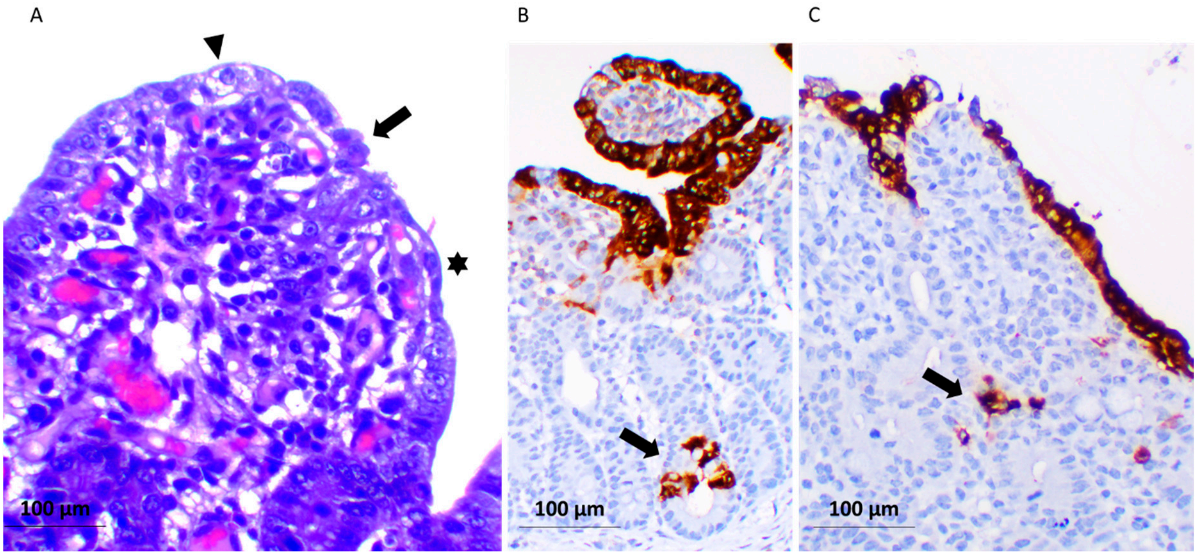


**Supplementary Figure S2.** (A) Epithelial damage and degeneration in PEDV-infected enterocytes at 48hpi. Hematoxylin and Eosin (H&E). The apical part of the atrophic and fused villi is lined either by a vacuolated cuboidal epithelium (degeneration-arrowhead) or by an attenuated epithelium (asterisk) characterized by cellular retraction with cytoplasmic hypereosinophilia and nuclear pyknosis (necrosis) (arrow). (B and C) Scattered PEDV-Immunolabeling cells were also observed in few intestinal glands (Crypts of Lieberkühn) (arrow- B) and in dendritic-like cells of the intestinal lamina propria (arrow- C).
